# From Atoms to Colloids: Does the Frenkel Line Exist in Discontinuous Potentials?

**DOI:** 10.1021/acsomega.2c08056

**Published:** 2023-03-23

**Authors:** Ciprian G. Pruteanu, Marcus N. Bannerman, Marcin Kirsz, Leo Lue, Graeme J. Ackland

**Affiliations:** † SUPA, School of Physics and Astronomy and Centre for Science at Extreme Conditions, The University of Edinburgh, Edinburgh EH9 3JZ, United Kingdom; ‡ School of Engineering, University of Aberdeen, Aberdeen AB24 3UE, United Kingdom; § Department of Chemical and Process Engineering, University of Strathclyde, James Weir Building, 75 Montrose Street, Glasgow G1 1XJ, United Kingdom

## Abstract

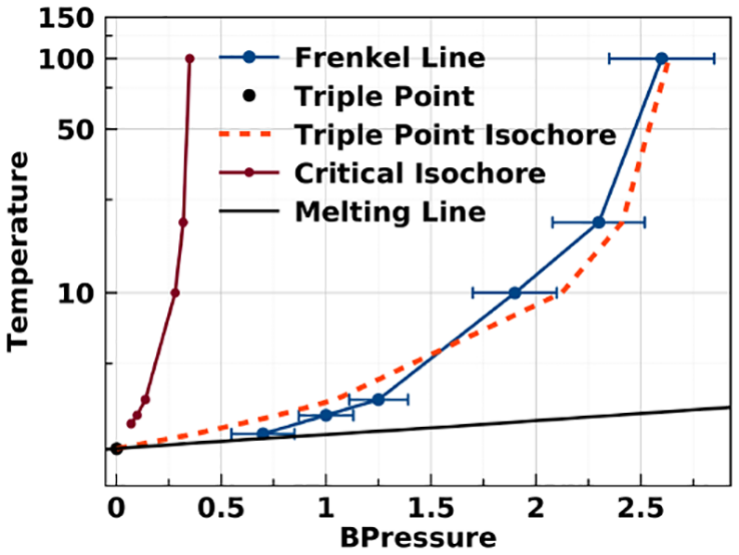

The Frenkel line has been proposed as a crossover in the fluid region of phase diagrams between a “nonrigid” and a “rigid” fluid. It is generally described as a crossover in the dynamical properties of a material and as such has been described theoretically using a very different set of markers from those with which is it investigated experimentally. In this study, we have performed extensive calculations using two simple yet fundamentally different model systems: hard spheres and square-well potentials. The former has only hardcore repulsion, while the latter also includes a simple model of attraction. We computed and analyzed a series of physical properties used previously in simulations and experimental measurements and discuss critically their correlations and validity as to being able to uniquely and coherently locate the Frenkel line in discontinuous potentials.

## Introduction

About a decade ago, a proposal was put forward that within the supercritical fluid region of a phase diagram a clear crossover exists between a “nonrigid” and a “rigid” fluid.^1^ This crossover, now referred to as the “Frenkel line”, occurs at higher pressures than the vapor curve in the vicinity of the critical point and continues into the supercritical regime. It is different from the Widom lines which start at the critical point. Widom lines represent maxima or minima in thermodynamic properties which diverge at the phase line, such as heat capacity or compressibility. The Frenkel line is related to dynamic and structural properties such as the velocity autocorrelation function, diffusion constant, and coordination number.

The initial concept of the Frenkel line focused on dynamical behavior and so proposed different dynamics-related observables in order to locate it. Specifically, the diffusion coefficient may change when crossing the Frenkel line due the mean free path becoming shorter than the molecular diameter. Above the Frenkel line densities, there is likely to be a change in the primary mechanism of diffusion from collective motion of atoms/molecules at lower densities to individual atomic/molecular movement between cages formed by tightly packed nearest neighbors. This caging effect makes it possible for the liquid to support a high-frequency shear wave: another proposed signature of the Frenkel line.

Another property related to neighbor caging is the velocity autocorrelation function (VACF), *C*(*t*) = ⟨**v**(0)·**v**(*t*)⟩. In a free-flowing fluid, the VACF decays to zero at a rate which increases with the number of collisions and therefore the density. By contrast, a caged atom/molecule may oscillate, leading to a region of negative VACF. The existence of a negative region, or a turning point, in the VACF is another candidate parameter mapping the Frenkel line. A notable exception occurs for hard-sphere systems,^2^ where even in the crystalline form there is no temperature-independent characteristic frequency that could appear in the VACF. On this basis, some authors have argued against the meaningfulness of the Frenkel line in itself.^3^

Another proposed signature is the minimum in the Raman frequency along an isochore.^4^ This is well defined for molecules with a single Raman mode, although useless for atomic fluids and ambiguous where there are several molecular modes. The idea here is that the changeover comes between the lowering of the frequency due to long-range coupling between molecules and the increase in frequency due to short-range repulsion.

Finally, the Frenkel line may have a signature in the thermodynamic properties which are derivatives of the free energy.^5^ Of these, the equation of state, heat capacity, thermal expansivity, and compressibility are the easiest to determine. These criteria have been successfully applied in soft-sphere and Lennard–Jones models, which were the first systems in which the idea of a Frenkel line was explored.^6^ For the specific case of heat capacity, a clear-cut *C*_*V*_ = 2*k*_B_ criterion was put forward to represent the Frenkel line. As with the VACF, this criterion is undefined for the hard-sphere system, pointing to hard spheres being a pathological outlier case.

Unfortunately, while the quantities mentioned above may be readily accessible in calculations and simulations, they are prohibitively challenging to measure experimentally to the accuracy required to determine such subtle changes.

On the other hand, structural measurements are much more practically available and widespread.^7^ These do offer the necessary accuracy and precision for the detection of subtle crossovers.^8^ Several studies have correlated some of the dynamic criteria for the Frenkel line, such as the minimum in Raman frequency, to structural changes in nitrogen^9,10^ and neon.^11^

For example, the coordination number for each atom/molecule plateaus at about 12 neighbors. The coordination number can be defined both precisely and arbitrarily from the radial distribution function *g*(*r*)—normally as the mean number of neighbors closer than the first nonzero minimum in *g*(*r*). The *g*(*r*) is an experimentally measurable quantity obtained from the structure factor *S*(*q*) measured in diffraction experiments. Calculating the coordination number introduces two sources of error, namely, counting the number of neighbors and determining the minimum in *g*(*r*) which defines whether a nearby atom counts as a neighbor or not. However, as it is defined, it is obvious that the coordination number should be proportional to density at low pressures and tend asymptotically toward the close-packing limit—12 for identical hard spheres—at high pressure. Although the change in coordination number with pressure is not sharp but continuous with pressure/density increase, the previously mentioned studies have related them to the Frenkel line.

A more recent study successfully reproduced the experimentally determined curves for nitrogen using a machine-learned classical force field^12^ and showed that the changes in coordination number correlate strongly with changes in diffusion coefficient. The latter mirrors the behavior of the coordination number and decreases continuously with increasing pressure/density, yet the same qualitatively different regions can be identified as for the coordination number.

The situation presented above shows a good level of consistency and coherence between what is theorized about the Frenkel line and what is experimentally determined. However, the current picture still leaves open a couple of fundamental questions concerning complex fluids: Do such fluids also have a Frenkel line? If not, what are the minimum features of an atomic/molecular potential that will lead to the presence of a Frenkel line and its consistent identification?

Here, we tackle this by applying all of the previously used criteria to two simple models, hard spheres and square-well potentials. The difference between these models is a fundamental one: hard spheres have zero potential energy, so the phase behavior depends only on one independent variable (e.g., density), and the Frenkel “line” becomes a point. The square-well attraction means both the density and the temperature affect the macroscopic behavior.

Looking at simplified models allows us to understand what essential elements are required to reproduce key features and properties of “real” molecular systems.

## Description of Square-Well Systems

The square-well potential provides a simple and elegant model that captures the key physics of the interaction between molecules in real fluids, with the hard-sphere interaction preventing the direct overlap of the molecules and the square-well interaction describing the cohesive force that attracts molecules together. Within this model, particles interact with each other via the pairwise potential
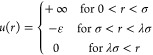
1where *r* is the distance between the particles. This describes hard spheres with diameter σ that interact via an attractive square well of depth ε and range *λσ*.

The hard-sphere system appears as three limiting cases of eq 1: (i) ϵ = 0, (ii) λ = 1, or (iii) 1/λ = 0. It does not have a liquid–gas transition, but it exhibits an entropy-driven solid–fluid transition, with a freezing density of ρ_f_σ^3^ ≈ 0.943, a melting density of ρ_m_σ^3^ ≈ 1.041, and a maximum density of ρ_cp_ = √2 when it is in the face-centered cubic close-packing limit. Extrapolation of the fluid phase into the metastable region leads to the pressure divergence at random close packing ρ_r_σ^3^ ≈ 1.23.

To obtain a liquid–gas transition, it is necessary to have attractive interactions between particles. The square-well potential (eq 1) with λ = 1.5 was one of the first to be studied by molecular dynamics;^13^ it exhibits gas–liquid, liquid–solid, gas–solid, and solid–solid phase transitions. It is arguably the simplest model to capture this full range of possibilities, and as a consequence, it has played a key role in the development of the theory of the structure, thermodynamics, and dynamics of fluids.

The model has been found to give a good description of the thermodynamic properties of experimental fluids, and many free energy models based on the square-well interaction potential have been developed, most notably the statistical associating fluid theory (SAFT),^14−16^ which are used in practical engineering design calculations. Force field models for molecular simulations have also been developed based on square-well potentials.^17^ Slight modifications of the potential, such as adding additional “steps”, have been found to provide a good representation of a wide variety of molecules.^18,19^

Based on comparison of the kinetic theory results with experimental data for the transport properties, specifically the self-diffusion coefficient viscosity and thermal conductivity of noble gases, it was found that the square-well potential offered a good representation of the dynamics of experimental fluids.^20−22^ These theoretical expressions form the basis for the correlation and prediction of the viscosity of experimental fluids.^23^

The square-well model has also been used to describe the properties of colloidal systems,^24−26^ in particular protein solutions. The thermodynamics of aqueous protein solutions was found to be well described by the behavior of square-well fluids.^27,28^ Square-well fluids also provide a good model for the dynamics of these systems.^29,30^

Due to its simplicity and the important role it plays, there is a large body of molecular dynamics and Monte Carlo simulation data available for the thermodynamic properties,^31^ phase behavior,^32−35^ and transport properties^36^ of square-well fluids of varying well width λ. In this work, we examine square-well systems with λ = 2. Results for the phase behavior are given in the following section.

## Results

### Phase Behavior

To determine the vapor–liquid coexistence region, multicanonical Monte Carlo simulations were performed^33,37,38^ in cubic, periodic simulation boxes of side length *L* = 7σ, 10σ, 12σ, and 15σ. The weights of the insertion and deletion of the square-well particles were adjusted in order to obtain uniform sampling of the number of particles in the system. From these weights, the point of vapor–liquid coexistence was determined by finding the chemical potential at which the probability of the system being in the vapor phase equaled the probability of it being in the liquid phase.

**Figure 1:**
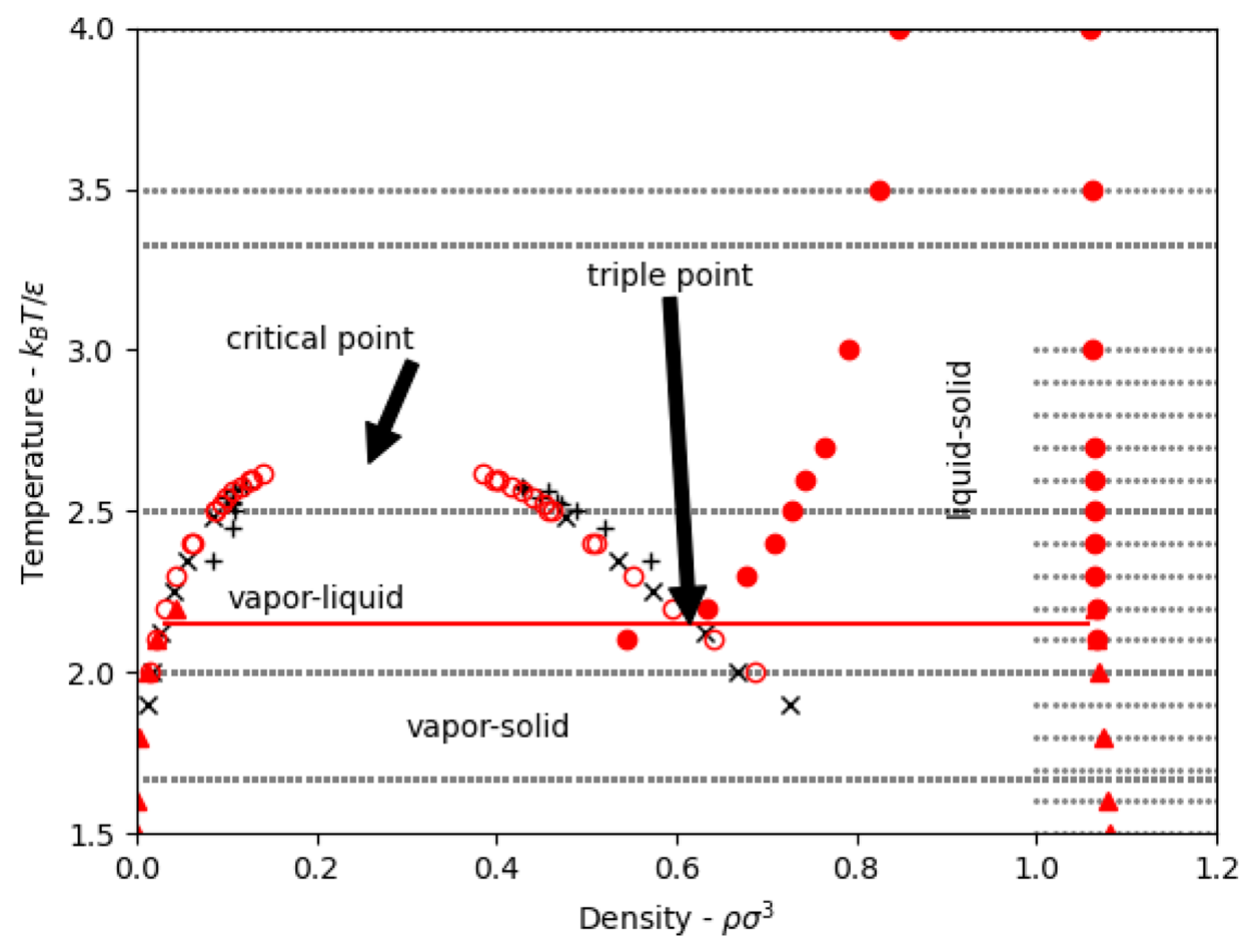
Temperature–density phase diagram for square-well systems with λ = 2. The open circles mark the vapor–liquid coexistence envelope, as calculated using multicanonical Monte Carlo simulations in this work; previous simulation results from Elliott and Hu^32^ are depicted as “×”, and calculations of Vega et al.^39^ are depicted as “+”. The red solid circles mark the solid–liquid coexistence region, as calculated using MD simulations in this work. The small black squares represent the data points calculated in the current study along different isotherms.

Our Monte Carlo results for the vapor–liquid-phase coexistence curve with λ = 2 are shown as the open red circles in Figure 1. We estimate the critical point to be located at *k*_B_*T*_c_/ε ≈ 2.664 ± 0.002 and ρ_c_σ^3^ ≈ 0.258 ± 0.002. Our predictions for the coexistence curve compare well with the results of Elliott and Hu,^32^ who used Gibbs–Duhem integration (shown as black ×’s); they estimate the critical point to be *k*_B_*T*_c_/ε ≈ 2.61, *p*_c_σ^3^/ε ≈ 0.17, and ρ_c_σ^3^ ≈ 0.27, in agreement with the older work of Vega and co-workers,^39^ which used Gibbs ensemble simulations (shown as black +’s); they estimated the vapor critical point to be located at *k*_B_*T*_c_/ε ≈ 2.764 ± 0.023, *p*_c_σ^3^/ε ≈ 0.197 ± 0.026, and ρ_c_σ^3^ ≈ 0.225 ± 0.018

We determine the solid–liquid coexistence line using MD simulations at densities from *ρσ*^3^ = 1.0 to 1.4. In addition, simulations were performed along the isochore *ρσ*^3^ = 1.3 for temperatures from *k*_B_*T*/ε = 1 to infinity (the hard-sphere limit). The free energy of the solid *fcc* phase could then be integrated with respect to the hard-sphere system, which was calculated using the equation of state of the hard-sphere solid taken from ref (40), and the residual Helmholtz free energy at *ρσ*^3^ = 1.21 is 7.984 ± 0.001.^41^

Based on the intersection of the vapor–liquid and solid–liquid coexistence curves, the triple point is estimated to be ρ_t_σ^3^ ≈ 0.61, *k*_B_*T*/ε ≈ 2.16, and *p*_c_σ^3^/ε ≈ 0.056. Our melting curve is in good agreement with previous estimates using the cell model.^42^

### Equation of State and Event Rates

**Figure 2:**
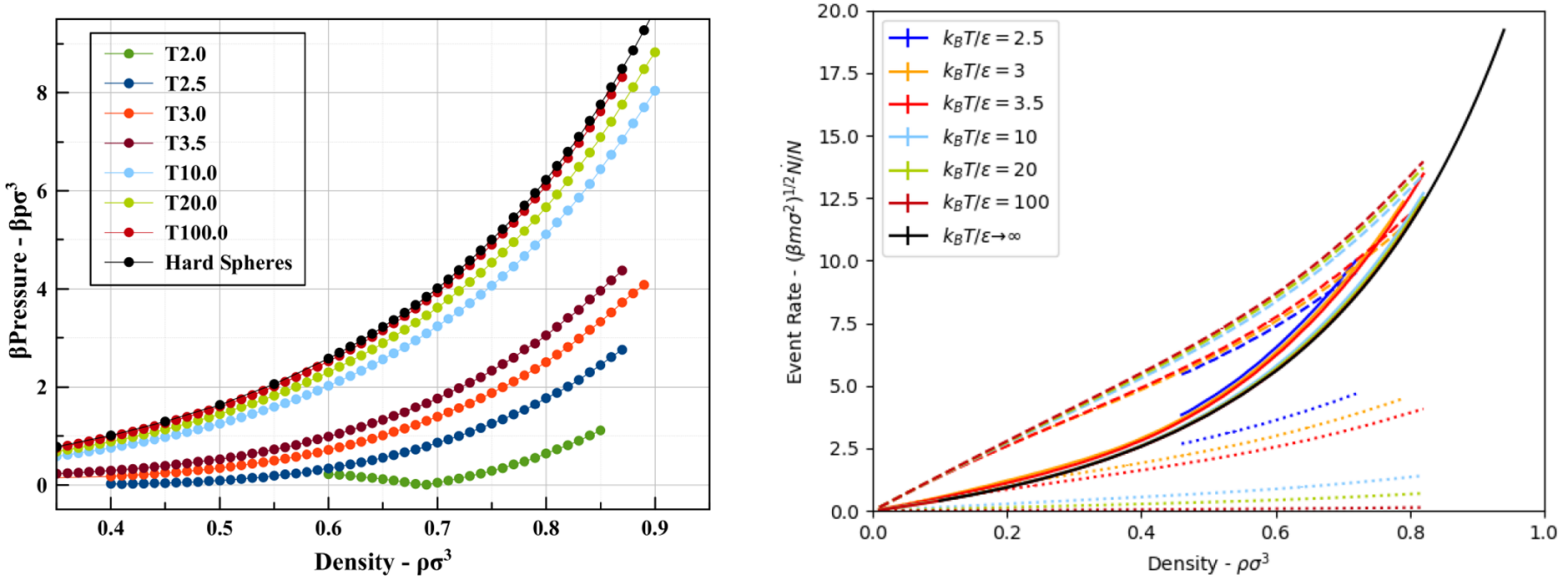
(Left) Equations of state, as βP vs density (*ρσ*^3^) along the isotherms followed in the present study. The *T* = 2.5 *k*_B_/ϵ isotherm shows an anomaly (discontinuity and pressure decrease on density increase) due to crossing the vapor–liquid coexistence region at the lower densities. (Right) Event rates for square-well systems with λ = 2 at a temperature *k*_B_*T*/ε = 2.5 (blue), *k*_B_*T*/ε = 3 (orange), *k*_B_*T*/ε = 3.5 (red), and *k*_B_*T*/ε → ∞(black). The solid lines represent the core events, the dashed lines represent the capture/disassociation events, and the dotted lines represent the bounce events.

Event-driven MD simulations are performed using the DynamO^43^ code to determine the structural, thermodynamic, and dynamic properties in the one-phase region. The equation of state is shown in Figure 2.

For single-component square-well systems, there are four types of events:^44^ (i) core events, where the hard-sphere portions of two particles collide, (ii) capture events, where two particles enter each others’ interaction well, (iii) disassociation events, where two particles exit each others’ square wells, and (iv) bounce events, where two particles moving away from each other are reflected back due to the square-well interaction. At equilibrium, only two of the rates for the four different events are independent. Only capture and disassociation events change the energy, so their rates must be equal. The rate of bounce events  is
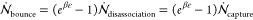
2In addition, the pressure of a square-well system can be directly related to the event rates^44^
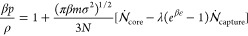
3The rates of the core and disassociation/capture events for the square-well systems along different isotherms are shown in Figure 2.

In the following sections, we examine various criteria for the Frenkel line for square-well fluids with λ = 2. We begin with the heat capacity and then move on to the VACF. We then examine the diffusion coefficient. Finally, we define a coordination number and describe its use in locating the Frenkel line. The various criteria for the Frenkel line are then compared to assess their consistency in determining its location.

### Energy and Heat Capacity

**Figure 3:**
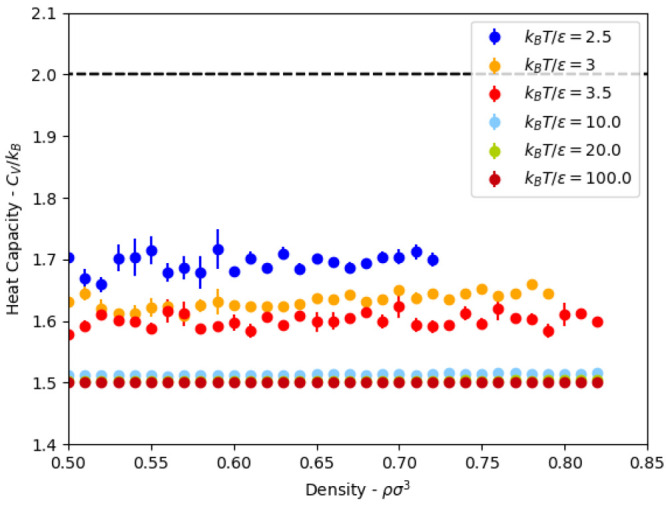
Isochoric heat capacity for square-well fluids with λ = 2.0 for all *k*_B_*T*/ϵ ratios investigated in the present study.

The isochoric heat capacity is shown in Figure 3 and is in agreement with previous simulations.^31^ Above the critical temperature, the heat capacity has a peak with respect to density, which denotes the Widom line; this lies at lower density than our simulations. In all of our simulations, *C*_*V*_ < 2*k*_B_, with the heat capacity increasing as *T* is lowered and the attraction becomes more relevant. More importantly, there is no discernible distinguishing feature with the pressure increase/volume change, making the heat capacity an ineffective parameter for locating the Frenkel line: this follows from the Frenkel line being about dynamic rather than structural properties. A similar lack of feature can be seen in the compressibility (slope of EoS, Figure 2).

This is expected as the heat capacity of hard-sphere fluids is exactly 3/2*k*_B_, which corresponds to the infinite temperature limit of the square-well fluid, and the potential provides one additional degree of freedom. This implies that the Frenkel line will be undetectable with the heat capacity criterion at a sufficiently high temperature for the square-well fluid.

### Coordination Number

Within the literature of square-well potential fluids, the coordination number is typically defined as the total number of particles within the attractive well (less than *λσ* apart). In this case, the energy per particle is in fact the average coordination number, times – ϵ. There has been work on developing models for the coordination number of square-well fluids.^45−47^ This is found to vary approximately linearly with density, with a slightly negative curvature for most well widths; interestingly, however, this curve has a positive curvature for small well widths (λ ≲ 1.1).^47^ A preliminary observation is that there does not seem to be a clear change in the slope of the variation of this definition of the coordination number with density for square-well fluids. So, this criterion seems to discount the presence of a Frenkel line for these systems. On the other hand, the first coordination shell of the hard-sphere system levels off at around *ρσ*^3^ = 0.9.^48^ Note that the liquid transition density is *ρσ*^2^ ≈ 0.94. Compressibility also appears to converge above *ρσ*^3^ = 0.9.

**Figure 4:**
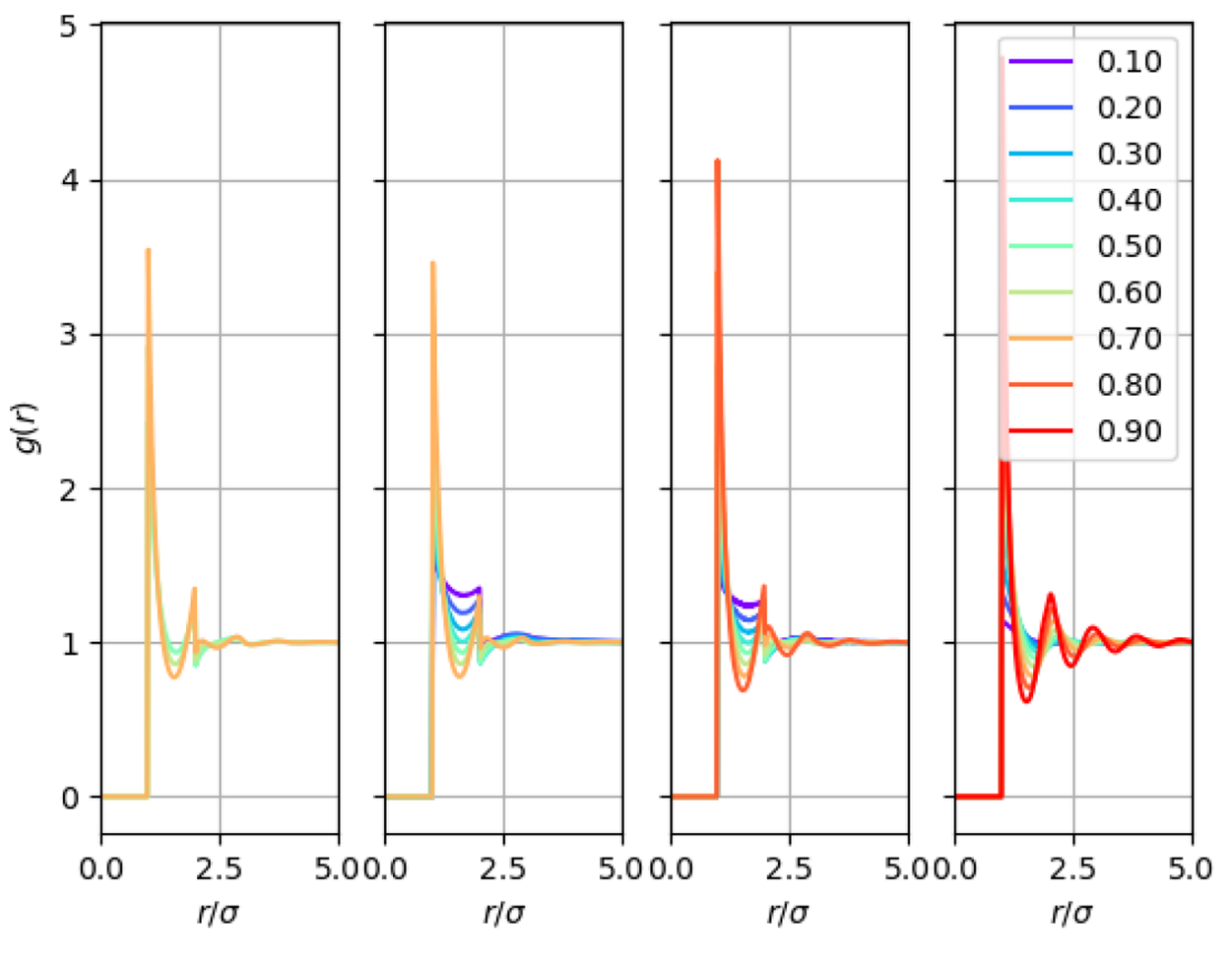
Radial distribution function for square-well fluids at *k*_B_*T*/ε = 2.5, 3, 3.5, and → ∞(i.e., hard spheres). The lines are labeled according to their density, *ρσ*^3^.

**Figure 5:**
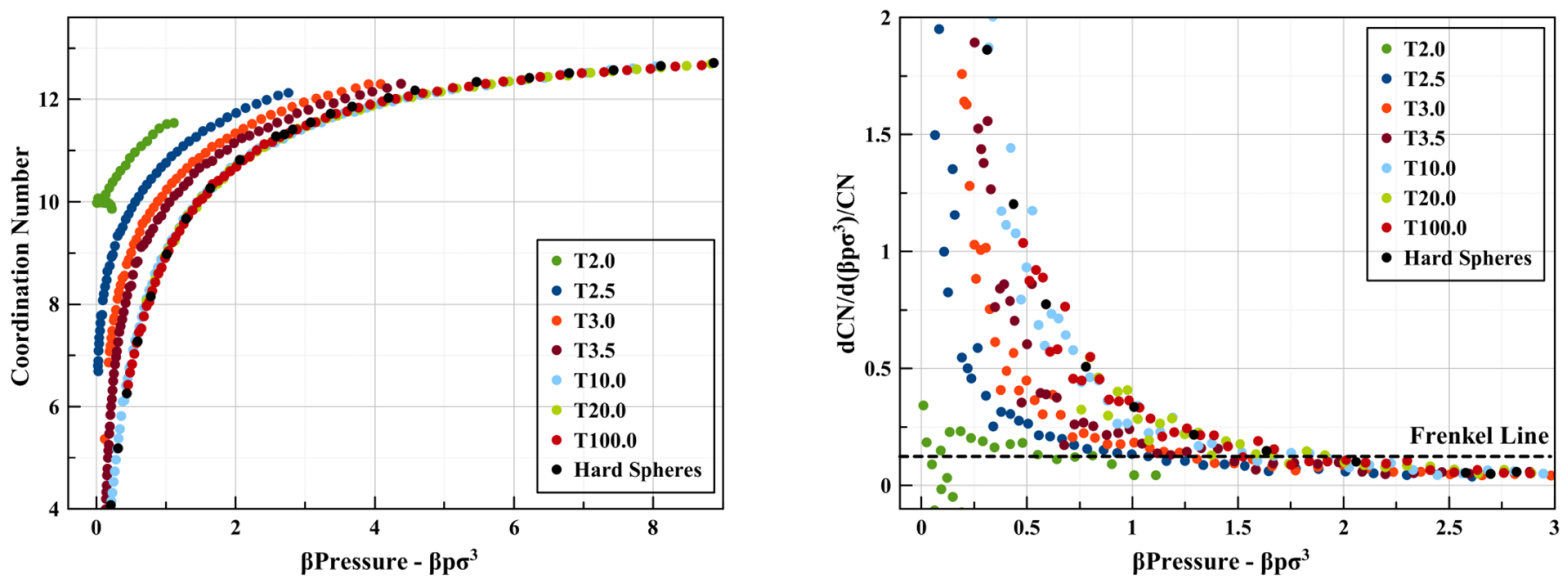
All coordination number-related results for λ = 2 square wells and hard spheres. (Left) Coordination number vs pressure, CN vs β*P*. (Right) d(log(CN))/d(β*P*) vs β*P*.

In contrast, in structural studies concerning real liquids, the coordination numbers are customarily obtained by integrating the respective pairwise radial distribution functions up to the first nonzero minimum, as done in nitrogen and krypton measurements by Pruteanu et al.^9,10,49^ The radial distribution functions for the square-well fluids are shown in Figure 4. It is readily visible for all temperatures that there is a discontinuity at *r*/σ = λ = 2, which is characteristic of the square-well fluid. Fortunately, the first minimum falls below this distance for all conditions explored in the present study; hence, the discontinuity does not influence the integral to obtain coordination numbers as its upper limit is always below *r*/σ = λ = 2. The coordination numbers extracted from the *g*(*r*)’s shown above as a function of *βP* are presented in Figure 5 (we use “beta pressure” in order to compare with the hard-sphere limit). A similar trend to that seen in nitrogen is readily visible here as well: at lower pressures/densities, the coordination numbers increase almost linearly with pressure and show a tendency to flatten asymptotically to 12 as pressure is increased. The log variation of the coordination number was calculated, and the square-well fluids were found to obey a similar criterion to the Pruteanu–Ackland^12^ one for nitrogen. The analytical and general form of the criterion (in dimensionless quantities) is presented in the following sections.

### Velocity Autocorrelation Function

**Figure 6:**
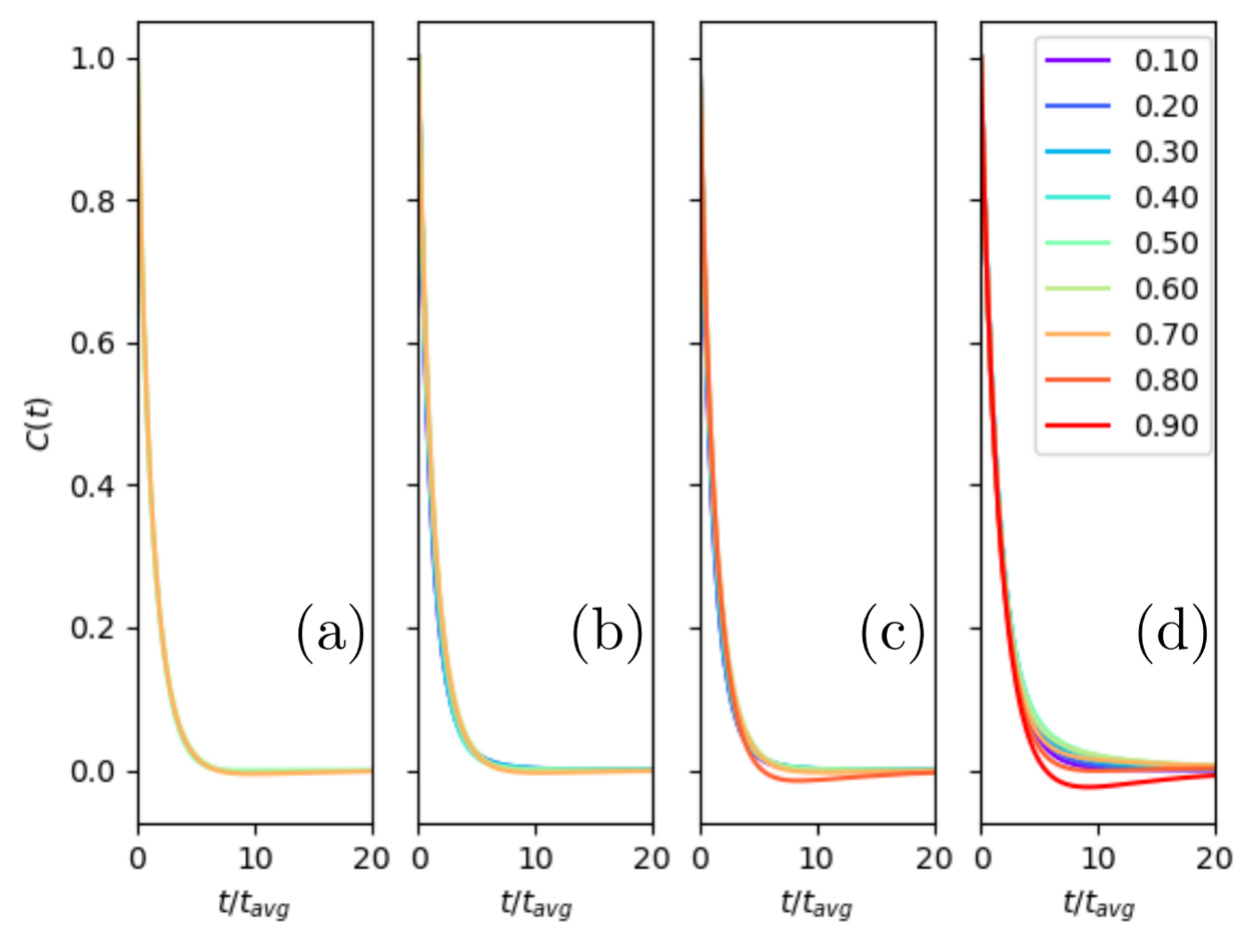
Velocity autocorrelation function for square-well fluids above the critical temperature: (a) *k*_B_*T*/ε = 2.5, (b) *k*_B_*T*/ε = 3, (c) *k*_B_*T*/ε = 3.5, and (d) *k*_B_*T*/ε → ∞ (hard-sphere limit). The lines are labeled according to their density, *ρσ*^3^.

The velocity autocorrelation function *C*(*t*) is defined as
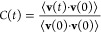
4where **v**(**t**) is the velocity of a particle at time *t*. To calculate the velocity autocorrelation function, the particle velocities were sampled with a time interval equal to one-tenth of the mean time *t*_avg_ between collisions for a hard-sphere system at the same density, as estimated by the Carnahan–Starling equation.^50^ It is plotted for the square-well fluids at several different temperatures in Figure 6.

**Figure 7:**
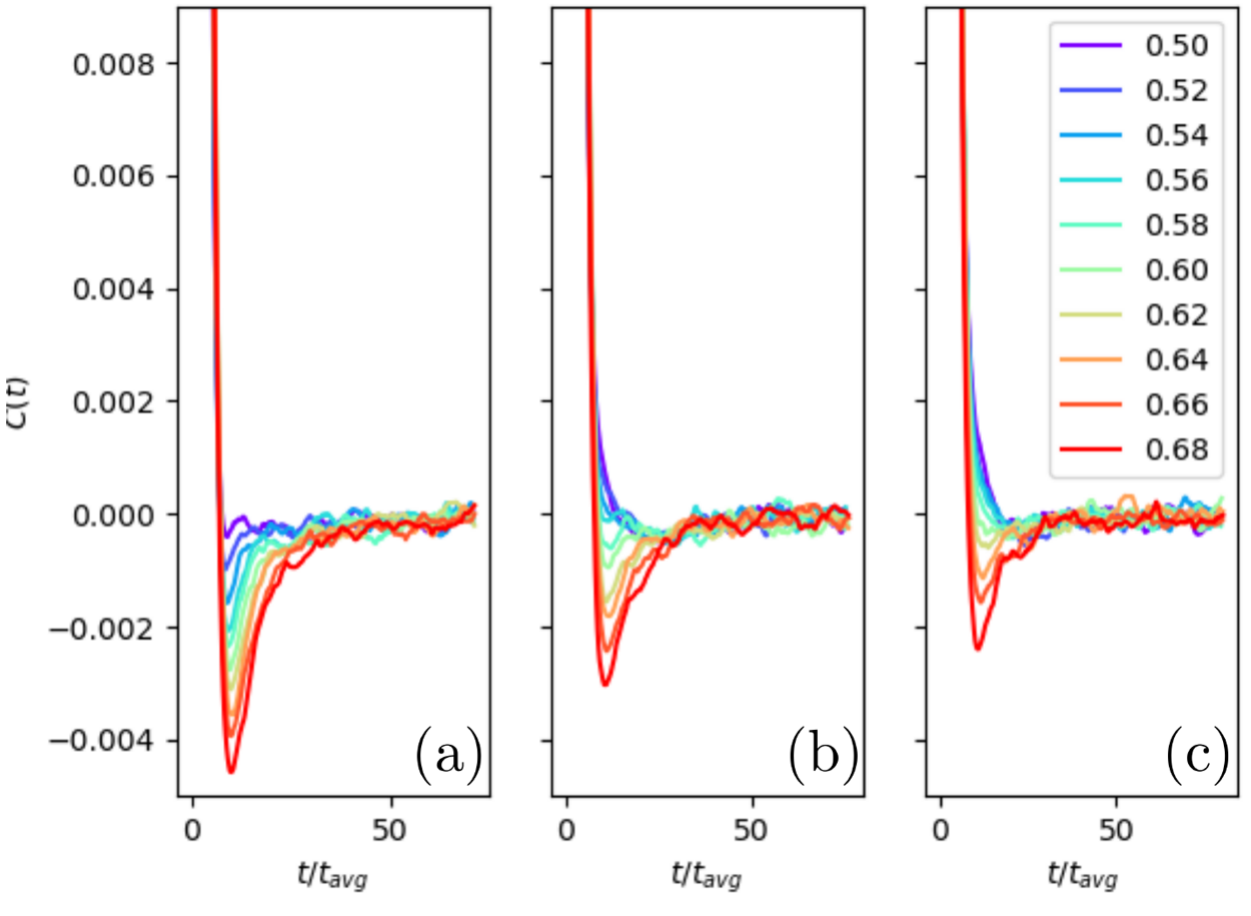
Plot of the velocity autocorrelation function of square-well fluids at (a) *k*_B_*T*/ε = 2.5, (b) *k*_B_*T*/ε = 3, and (c) *k*_B_*T*/ε = 3.5. The lines are labeled according to their density, *ρσ*^3^.

At low densities, the VACF monotonically decreases from its maximum value of 1 to a minimum value of 0 at large times, Figure 7. As the density increases, the autocorrelation function begins to develop a minimum. This implies a characteristic time, which could arise from either capture by the attractive potential (bouncing events) or caging effects. At higher densities the VACF has a negative region, implying the caging affects the majority of particles. These are signatures of the Frenkel line, but they are only visible in the VACF if they have some characteristic time scale.^51^ For soft potentials, this time may represent “almost harmonic” shear modes with a frequency related to the excitation energy ℏω. For square wells, there is no curvature to provide a distinctive frequency. However, the caging effect and rattling modes still exist in this extreme anharmonic limit.

**Figure 8:**
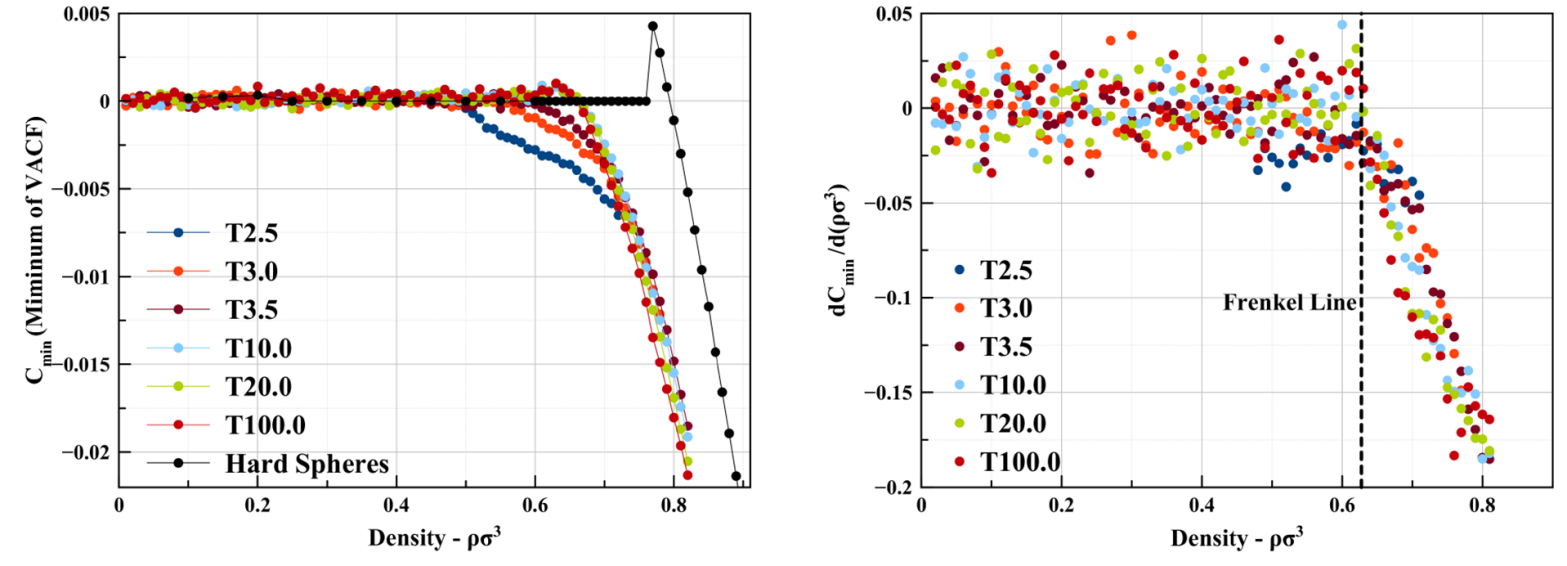
(Left) Plot of the (local) minimum value of the velocity autocorrelation function, *C*_min_(*t*), shown for a range of temperatures as a function of density. (Right) Variation of the minimum of the velocity autocorrelation function with density, d*C*_min_(*t*)/d(*ρσ*^3^).

In Figure 8, we plot the value of the first local minimum of the VACF as a function of density (or pressure) along different isotherms. Note that the hard-sphere system corresponds to the limit *T* → ∞.

### Diffusion Coefficient

**Figure 9:**
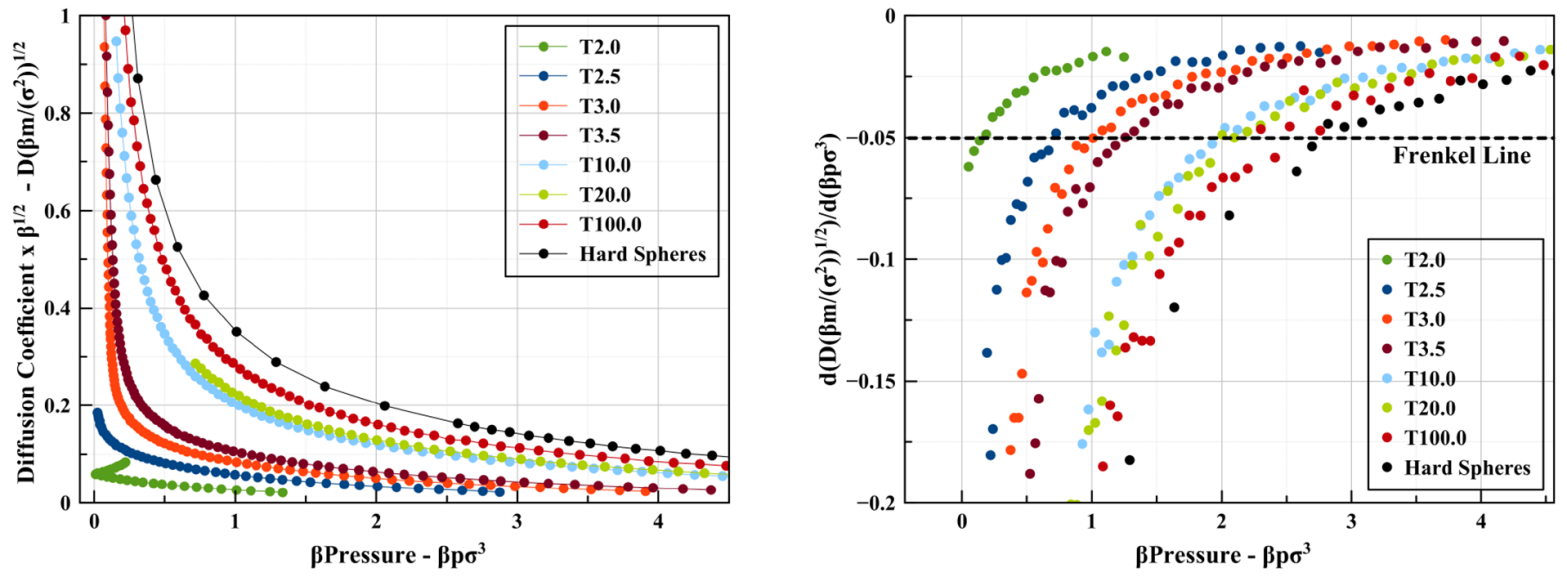
All diffusion constant-related results for λ = 2 square wells and hard spheres. (Left) Diffusion coefficient vs pressure, *D*/√*T* vs β*P*. (Right) Variation of the slope of the diffusion coefficient with pressure, d(*D*/√*T*)/d(β*P*) vs β*P*.

The integral of the VACF gives the diffusion constant via a Green–Kubo relationship
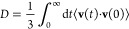
5The variation of the diffusion coefficient with density is shown in Figure 9.

The diffusion coefficient changes continuously with increasing β*P* throughout the whole range studied for all considered temperatures. Looking its the evolution of its slope with pressure, we can define (by correlation to the coordination number) a point where this quantity equals −0.05 for the location of the Frenkel line.

### Criteria for Frenkel Line Based on Results Above

Due to the relative simplicity of square-well and hard-sphere systems, we were able to correlate the various changes in different parameters and have managed to obtain a set of general (dimensionless) equations for the location of the Frenkel line on a phase diagram. The criteria were as follows.

- Heat capacity: does not correlate as it is fixed for classical hard spheres and almost constant for square-well fluids.
- Velocity autocorrelation function:
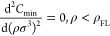

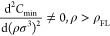
- Coordination number:
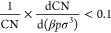
- Diffusion coefficient:
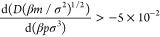

Applying these criteria to our current simulations of square wells and hard spheres leads to the locations for the Frenkel line in Table 1.

**Table 1:** Location of the Frenkel Line with λ = 2 According to the Criteria Named in the Present Study

system	*k*_B_*T*/ϵ	βPres (CN + D)	βPres (VACF)	*ρσ*^3^ (CN + D)	*ρσ*^3^ (VACF)
square wells	2.0				
square wells	2.5	0.70(15)	0.61(15)	0.68(2)	0.66(2)
square wells	3.0	1.00(13)	1.08(15)	0.65(2)	0.66(2)
square wells	3.5	1.25(14)	1.32(14)	0.64(2)	0.65(2)
square wells	10	2.02(20)	2.12(20)	0.60(2)	0.61(2)
square wells	20	2.41(22)	2.52(22)	0.61(2)	0.62(2)
square wells	100	2.60(25)	2.88(25)	0.61(2)	0.63(2)
hard spheres	∞	2.70	6	0.61	0.78

Two important observations are warranted at this point. The variation of the coordination number with density along the different isotherms becomes noticeably smoother as the temperature increases. As a consequence, the Pruteanu–Ackland criterion, as formulated, appears to struggle to identify the Frenkel line at temperatures significantly above the critical temperature, becoming more difficult to apply than the diffusion-based and VACF minimum criteria. On the other hard, while the coordination number- and diffusion coefficient-based criteria work for both square-well and hard-sphere systems, the VACF criterion does not. The original formulation of the VACF criterion (visible oscillatory behavior) correlates very well with the other criteria for square-well fluids, identifying the same location for the Frenkel line for most pressures/densities. It applies unequivocally for *T* ≫ *T*_c_ (3.76, 7.52, and 37.6 *T*_c_) but seems to underestimate the Frenkel line densities and pressures when compared to the coordination number and diffusion criteria for *T* ≈ *T*_c_ (0.94, 1.13, and 1.31 *T*_c_), where a more appropriate criterion appears to be a sudden change of slope of *C*_min_(*t*) with increasing pressure/density, once oscillations are already identifiable. At high densities, d*C*_min_/d*ρ* varies linearly with the density for all temperatures. As the density decreases, there is a kink in the variation of d*C*_min_/d*ρ* with density and its slope becomes zero. In contrast, in hard spheres, the VACF criterion would indicate significantly higher pressures/densities for the crossover, but as it has been noted by previous researchers, it should not be used due to the hard spheres being a pathological case.^2^ The origin of this discrepancy must be the existence of an attractive component.

## Discussion and Conclusion

The hard-sphere and square-well potentials have played a central role in the development of our understanding of the behavior of molecular fluids and colloidal suspensions. We find that both of these potentials produce Frenkel lines as identified by the correlated coordination number–diffusion coefficient criterion used in previous studies.^9,10,12,49^ The criterion was found to also correlate with changes in the velocity autocorrelation function, used for identifying the Frenkel line, at least for square-well potentials. The notable exception, as pointed by previous authors,^2^ is the hard-sphere system, where the velocity autocorrelation is uninformative as to the location of the Frenkel line, likely due to the describing potential being pathological by only containing repulsive interactions. The hard spheres, which have been interpreted on occasion as a limiting case of square-well potentials, in the limit *T* → ∞, can be viewed in this light in relation to the Frenkel line if the coordination number/diffusion coefficient criteria are solely employed. Moreover, the heat capacity criterion for the identification of the Frenkel line was found not to hold in either of our model systems, and hence, the behavior of the heat capacity can be ruled out as a fundamental one relating to the Frenkel line and the nonrigid to rigid fluid crossover.

**Figure 10:**
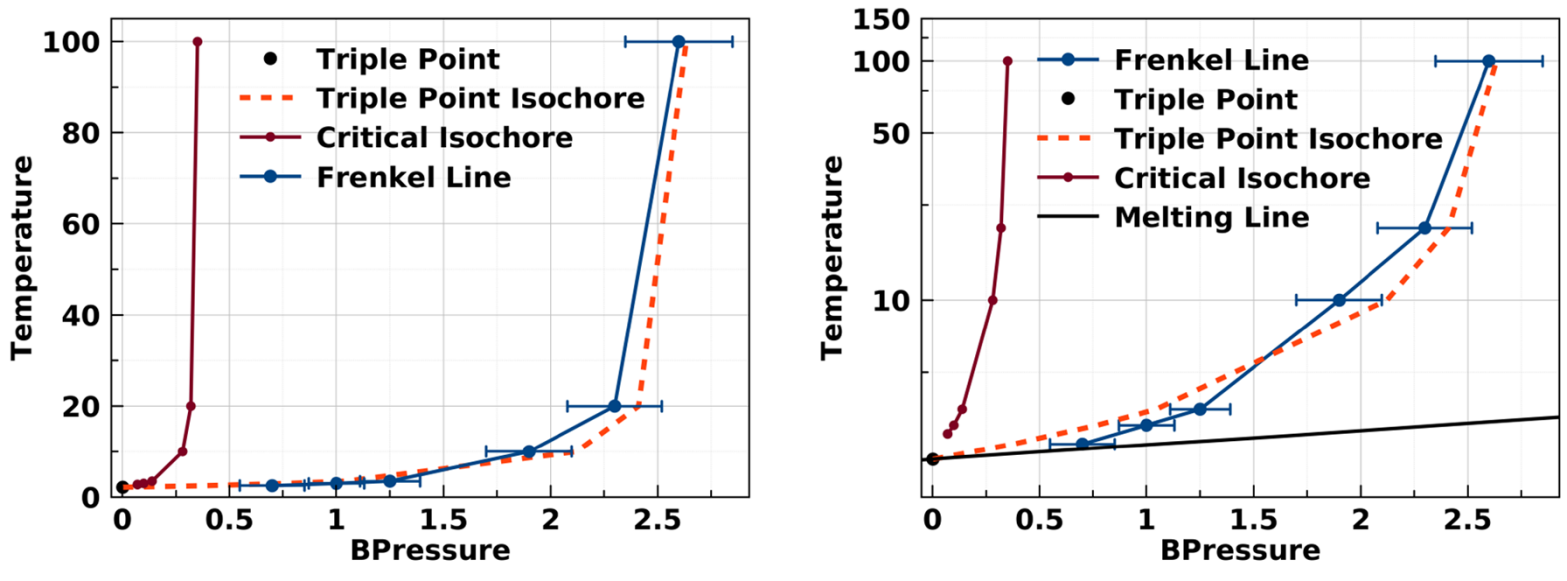
Liquid-side triple-point isochore, critical isochore, and Frenkel line for square-well fluids as identified in the present study. Temperature is in units of *k*_B_/ϵ, BPressure is *βpσ*^3^.

**Figure 11:**
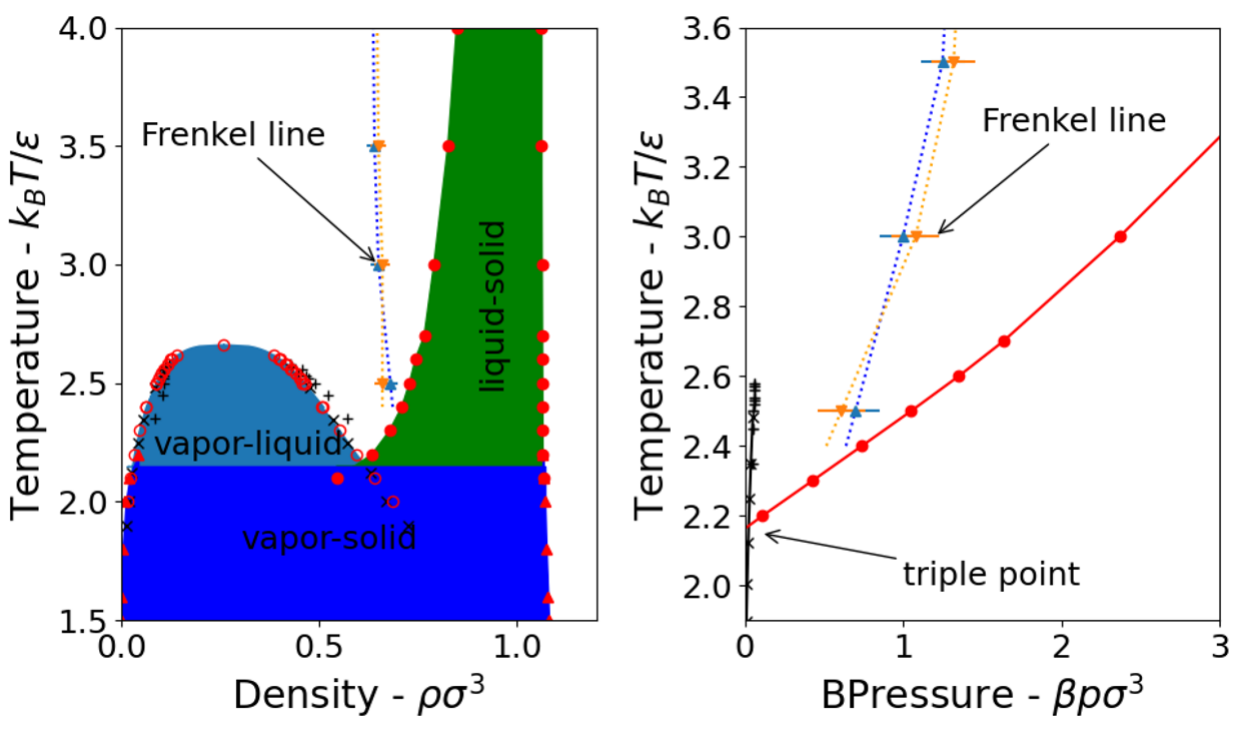
Location of the Frenkel line on temperature–density (left) and temperature–pressure (right) phase diagrams. In the *T*–*p* phase diagram, the black line is the vapor curve while the red one is the melting line. The upward triangles are the CN + D criterion, and the downward triangles are the VACF criterion.

A key finding of the present study is that the Frenkel line does seem to originate/terminate at the triple point, Figures 10 and 11, as previously proposed by Pruteanu et al.^12^ This provides the simplest and most clear-cut method for drawing the Frenkel line on any phase diagram, whether of real or model systems, by following the liquid-side isochore from the immediate vicinity of the triple point, i.e., the density of the liquid at the triple point. Based on this result and by correlating previously proposed disjointed theoretical and experimental criteria to uniquely locate the Frenkel line, we propose a set of quantitative generalized equations to determine the location of the Frenkel line in any given fluid.

We do note however a curious coincidence: for the particular choice of square-well width used in this study (λ = 2), the ratio *T*_TP_/*T*_c_ ≃ 0.8. This relative temperature (0.8 *T*_c_) was previously indicated as being the originating point of the Frenkel line on the boiling curve by Yang et al.^1,52^ Further studies involving different ranges (values of λ) of square-well potentials will be needed in order to resolve this matter definitively.

The Frenkel line tracks a crossover in behavior rather than any discontinuity: It occurs over a narrow pressure range. As a consequence, different manners of determining its location will lead to slightly different values. Hard spheres are often used as a model for colloidal suspensions: systems with completely nonharmonic interactions. This work shows how the Frenkel-line concept can be generalized to describe changes in dynamical behavior even in these types of discontinuous potentials.

The Frenkel line has similarities with the colloidal glass transition, which is also a crossover rather than a discontinuous transition. Both phenomena are density driven and related to changes in the dynamical behavior; in the case of the colloidal glass, the definition is usually in macroscopic viscosity rather than diffusion constant. A key difference is that the Frenkel line lies on the equilibrium phase diagram, while colloidal glasses are typically metastable and exhibit aging phenomena.
